# Cystic and Papillary Neoplasm at the Hepatic Hilum Possibly Originating in the Peribiliary Glands

**DOI:** 10.1155/2016/9130754

**Published:** 2016-08-31

**Authors:** Takashi Miyata, Katsuhiko Uesaka, Yasuni Nakanuma

**Affiliations:** ^1^Division of Hepato-Biliary-Pancreatic Surgery, Shizuoka Cancer Center, Shizuoka, Japan; ^2^Division of Diagnostic Pathology, Shizuoka Cancer Center, Shizuoka, Japan

## Abstract

Cystic neoplasms of the liver are divided into two types: mucinous cystic neoplasm and cystic intraductal papillary neoplasm of the bile duct. We herein report two cases of cystic and papillary neoplasm of the liver which differed from the abovementioned types.* Case  1.* A 70-year-old man. Radiologically, a cystic tumor measuring 20 mm in diameter was found at the hepatic hilum. Right hepatectomy was performed under a diagnosis of intrahepatic cholangiocarcinoma (iCCA) based on the imaging findings.* Case  2.* A 70-year-old man. Radiologically, a cystic tumor measuring 60 mm in diameter was found at the hepatic hilum. Under a diagnosis of iCCA, left hepatic trisectionectomy was performed. In both cases, endoscopic retrograde cholangiography did not demonstrate communication between the cystic tumor and adjacent bile ducts. Pathologically, these two tumors were cystic neoplasms located at the hepatic hilum and were morphologically characterized by an intracystic papillary neoplasm composed of diffuse high-grade dysplasia and associated with an invasive carcinoma. Ovarian-like stroma was not found in the capsule of these tumors. Interestingly, there were peribiliary glands near these tumors, and MUC6 was expressed in these papillary neoplasms as well as in the peribiliary glands. These neoplasms might have arisen from the peribiliary glands.

## 1. Introduction

The biliary tree is composed of the intrahepatic and extrahepatic bile ducts. The right and left hepatic ducts and the proximal portion of the extrahepatic bile duct are collectively called the “perihilar bile ducts.” The intrahepatic bile ducts, proximal to the branches of the right or left hepatic duct, are classified as intrahepatic large and small bile ducts [1]. The former consist of the second to third branches of the right or left hepatic bile ducts, while the latter consist of the septal and interlobular bile ducts. Cholangiocarcinoma (CCA) is usually classified into intrahepatic CCA, perihilar CCA, and distal type CCA in accordance with the anatomical location: distal CCA involves the common bile duct, perihilar CCA the perihilar bile duct, and intrahepatic CCA the branches of both hepatic ducts and more proximal bile ducts [2]. CCA arising in the hepatic parenchyma is an intrahepatic CCA. Recently, substantial progress has been made in the study of precancerous and preinvasive lesions of CCA: biliary intraepithelial neoplasm (BilIN), intraductal papillary neoplasm of bile duct (IPNB), and mucinous cystic neoplasm (MCN) [3–6].

According to the 2010 World Health Organization (WHO) classification of the digestive system, hepatic or biliary cystic neoplasms are classifiable into two categories: MCNs and cystic type of IPNBs [2, 3]. MCNs exclusively affect females and are characterized by mucin-positive lining epithelia and ovarian-like stroma in the cyst wall. MCNs do not communicate with the bile ducts. IPNBs develop in the perihilar bile ducts, including their major branches and the extrahepatic bile ducts, and are regarded to arise from the lining epithelia of the bile duct [2–4]. The affected ducts show cystic dilatation and often have mucus hypersecretion and direct communication with the adjacent bile ducts [2, 4–6]. IPNBs show papillary or villous growth of very differentiated neoplastic epithelia with delicate fibrovascular cores within the dilated bile duct lumen. MCN and IPNB show low-, intermediate-, and high-grade intraepithelial neoplasms (dysplasia), and some cases are associated with invasive carcinoma [2, 3].

The peribiliary glands are physiologically distributed around the intrahepatic large bile ducts and perihilar and distal bile ducts, and they drain into the lumen of bile ducts via their own conduits [1]. They are particularly densely located around the perihilar bile ducts. Thus far, several types of biliary neoplasms involving or arising from the peribiliary glands have been reported: cystic micropapillary neoplasm, biliary intraepithelial neoplasm, and CCA [7–13]. Nakanuma and Sato proposed that IPNB arising from the peribiliary glands be referred to as a branch type of IPNB [7]. Recently, Nakanishi et al. reported a case of cystic papillary neoplasm that might have arisen from the peribiliary glands and had cystic lesions that communicated with the bile duct lumen [12]. In addition, Sato et al. [13] recently reported an entity of cystic and micropapillary epithelial lesions of the peribiliary glands by surveying many autopsy cases; these lesions were characterized by grossly visible multicystic epithelial tumors with foci of micropapillary patterns. After an extensive analysis, they concluded that these lesions were neoplastic; therefore, the term “cystic micropapillary neoplasm of the peribiliary glands” may be used to indicate an incipient lesion of branch type IPNB. Uchida et al. also reported a cystic micropapillary neoplasm involving the peribiliary glands associated with hilar CCA [10]. However, the pathology and morphogenesis of this type of IPNB remain unclear.

We herein report two cases of intrahepatic cystic neoplasms located at the hepatic hilum that showed intracystic, well-differentiated papillary neoplasms composed of diffuse high-grade dysplasia with fine fibrovascular stroma and an associated invasive carcinoma, which might have originated from the peribiliary glands.

## 2. Case Reports

### 2.1. *Case  1*

A 70-year-old man with a history of hypertension and prostatic hypertrophy was admitted to our hospital with abdominal pain. Computed tomography (CT) revealed a round tumor measuring 20 mm in diameter in the right liver, and percutaneous liver biopsy identified adenocarcinoma.

**Figure 1:**
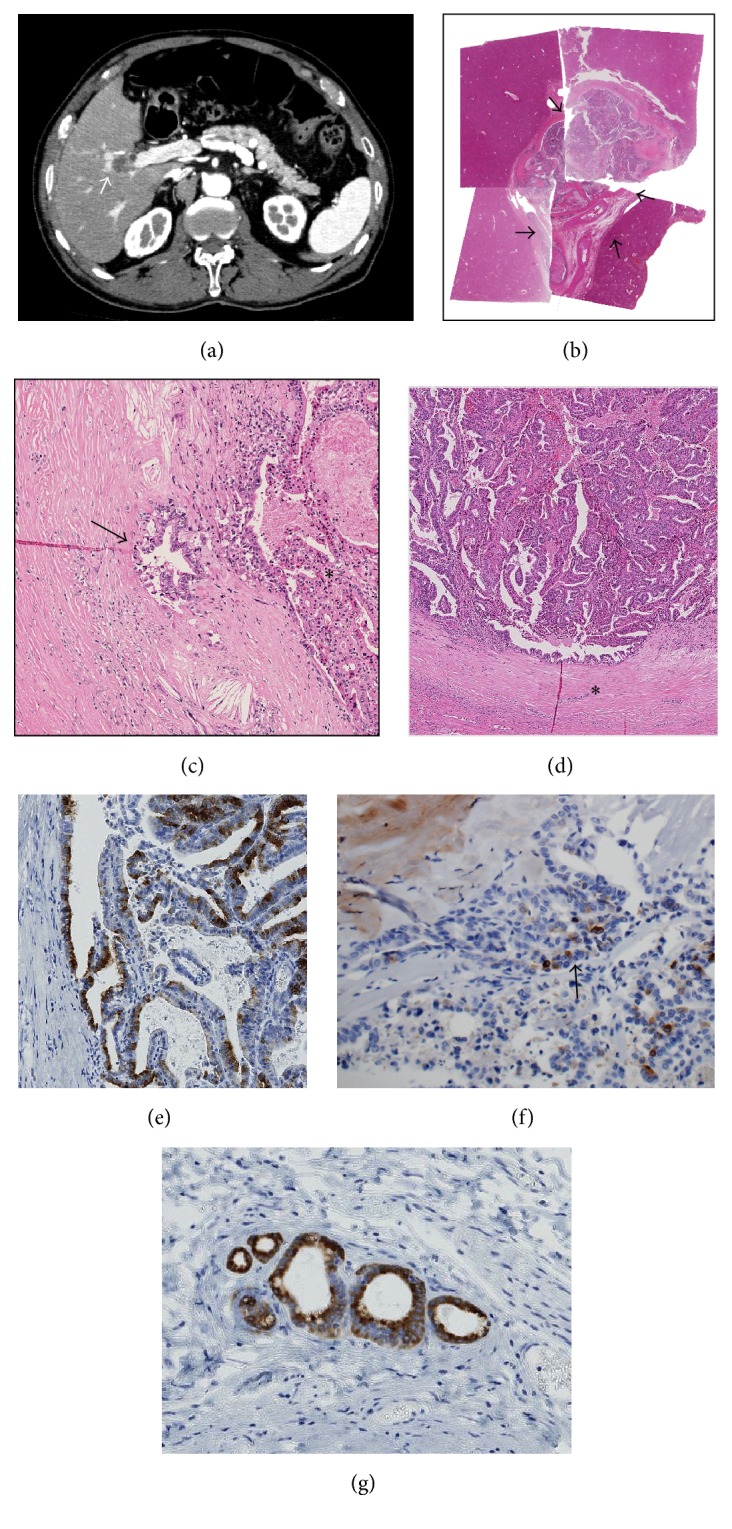
*Case  1.* (a) Contrast-enhanced CT shows a cystic lesion (arrow) (20 mm in diameter) at the confluence of the anterior and right bile duct with ring enhancement. There is no dilatation of the intrahepatic bile duct, except for slight dilatation of the proximal branches of the anterior branch of the bile duct. (b) The tumor (20 × 17 mm) is cystic and is filled with neoplastic cells and surrounded by a fibrous cyst wall (arrows). Loupe picture of the tumor (H&E staining). (c) Focal invasion of carcinoma is found in the fibrous capsule (arrow). Original magnification, 100x; H&E staining. (d) A papillary neoplasm composed of high-grade dysplasia with fine fibrovascular cores fills the cystic space of the tumor.  ^*∗*^ Fibrous capsule. Original magnification, 30x; H&E staining. (e) The papillary neoplasm is positive for MUC6. Original magnification, 100x; immunostaining for MUC6. (f) The invasive carcinoma in the fibrous capsule is positive for MUC6 (arrow). Original magnification, 100x; immunostaining for MUC6. (g) The peribiliary glands (nonneoplastic) adjacent to the tumor are also positive for MUC6. Original magnification, 100x; immunostaining for MUC6.

On admission, his general status was unremarkable. Regarding laboratory data, his blood cell counts and serum aspartate aminotransferase, alanine aminotransferase, and alkaline phosphatase levels were within the normal ranges. However, the levels of CEA and CA19-9 were elevated to 5.6 ng/mL (cut-off, <5.0 ng/mL) and 52 U/mL (cut-off, <37 U/mL), respectively. Abdominal enhanced CT detected a cystic lesion at the confluence of the anterior and posterior sectional duct with slight dilatation of proximal branches of the anterior sectional duct (Figure 1(a)). Endoscopic retrograde cholangiography (ERCP) revealed no communication between the cystic lesion and the bile duct. Right hepatectomy and lymph node dissection were performed under a diagnosis of intrahepatic CCA after percutaneous transhepatic portal embolization. The postoperative course for this patient was uneventful, and he was discharged. The carcinoma has not recurred in the past 24 months.

### 2.2. *Case  2*

A 70-year-old man was referred to our hospital due to liver dysfunction and a suspicion of gallbladder tumor. He had a history of diabetes mellitus and hypertension. A mass lesion was noted in the liver, and he was subsequently admitted to our hospital.

**Figure 2:**
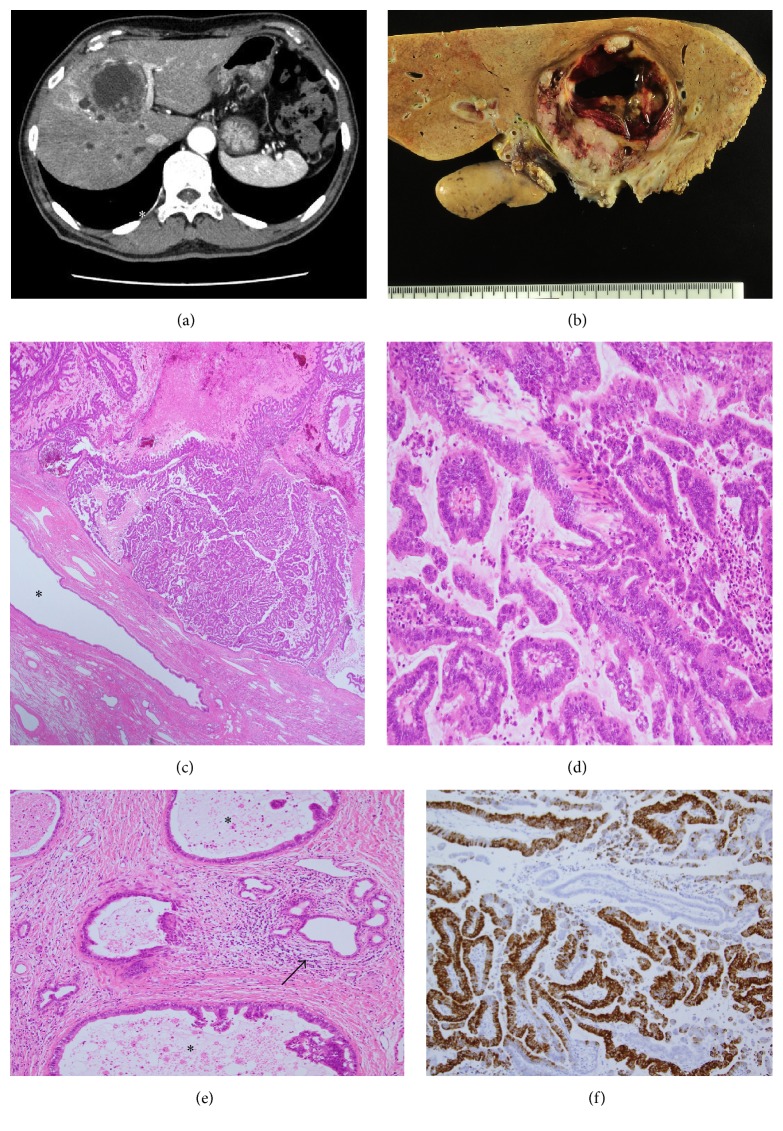
*Case  2.* (a) Contrast-enhanced CT shows a cystic lesion (60 mm in diameter) with an enhanced wall near the hepatic hilus. (b) A cystic tumor with fibrous wall is found in segment 4, adjacent to the hepatic hilus. (c) Histologically, well-differentiated papillary adenocarcinoma with thin fibrovascular cores fills the cystic space of the tumor. ^*∗*^ Surrounding nonneoplastic bile duct. Original magnification, 30x; H&E staining. (d) Papillary carcinoma with columnar epithelia and fine fibrovascular cores. Original magnification, 120x; H&E staining. (e) Invasion of carcinoma (*∗*) in the periductal tissue of adjacent bile ducts. The arrow denotes the nonneoplastic peribiliary glands. Original magnification, 100x; H&E staining. (f) Papillary carcinoma is positive for MUC6. Original magnification, 100x; immunostaining for MUC6.

On admission, his blood cell counts and serum bilirubin were within the normal ranges. However, his serum aspartate aminotransferase, alanine aminotransferase, and alkaline phosphatase levels were elevated to 88 (U/L), 150 (U/L), and 2090 (U/L), respectively. His CA19-9 levels were elevated to 42 U/mL at admission and 175 U/mL before surgical operation (2 months after admission) (cut-off, <37 U/mL), while his CEA levels remained approximately 2.0–2.4 ng/mL (cut-off, <5.0 ng/mL). Abdominal enhanced CT revealed a 60 mm cystic tumor mainly in segment 4, involving the hilar bile ducts (Figure 2(a)). However, ERCP revealed no communication between the cystic tumor and the adjacent bile ducts. The proximal intrahepatic bile ducts of the anterior and medial branch and medial segment branches were dilated. Under a diagnosis of intrahepatic CCA, left hepatic trisectionectomy was performed. The postoperative course for this patient was uneventful. The carcinoma has not recurred in the past 6 months.

### 2.3. Pathological Findings

#### 2.3.1. *Case  1*

Grossly, the tumor was a cystic lesion adjacent to the right hepatic duct and its anterior sectional duct. The cystic tumor, measuring 2.0 cm × 1.7 cm, was encapsulated by a fibrous capsule and filled with an exophytic papillary neoplasm and mucin (Figure 1(b)). Communication between the cystic tumor and adjacent bile ducts was not confirmed. Microscopically, the tumor showed a well-differentiated papillary neoplasm composed of high-grade dysplasia with cuboidal to columnar epithelial cells, acidophilic cytoplasm (focally, oncocytic), and delicate fibrovascular cores (Figures 1(b), 1(c), and 1(d)). Mucin detected by PAS after diastase digestion was observed in the cytoplasm of the neoplastic cells, and mucin hypersecretion was present. These neoplastic cells were histologically regarded as pancreatobiliary (PB) type with oncocytic type foci [2, 14]. The cyst wall was composed of dense fibrous tissue without ovarian-like stroma. While high-grade dysplastic cells were mainly confined in the cystic lumen, focal capsular invasion of carcinoma was observed (Figure 1(c)). We noted peribiliary glands and their conduits adjacent to this cystic tumor, particularly between the cystic tumor and the right hepatic duct (see Supplementaries 1 and 2 in Supplementary Material available online at http://dx.doi.org/10.1155/2016/9130754). Interestingly, we noted the intraepithelial spread of high-grade dysplastic cells through these glands and conduits, particularly their conduits; however, the right hepatic duct was spared from any neoplastic changes (Supplementary 2). Immunostaining of S100P, a marker of early cancerous or premalignant cells of the biliary tree and pancreatic ducts [10, 13], was strongly positive for the intracystic high-grade dysplastic cells as well as the intraepithelial-spreading dysplastic cells of the conduits of the peribiliary glands (Supplementary 3).

#### 2.3.2. *Case  2*

The cystic tumor, measuring 7.0 cm × 6.5 cm, was mainly located in segment 4, adjacent to the right hepatic duct and the posterior sectional duct. This cystic tumor was encapsulated by a fibrous capsule and filled with an exophytic papillary neoplasm, necrotic tissue, and mucin (Figure 2(b)). Communication between the cystic tumor and adjacent bile ducts was not confirmed. Histologically, this tumor showed a papillary neoplasm composed of high-grade dysplasia with fine fibrovascular stroma (Figures 2(c) and 2(d)). Phenotypically, the carcinoma was regarded to be the gastric type (Figure 2(d)) [1]. Although the tumor was surrounded by fibrous tissue, ovarian-like stroma was not observed. Invasion of carcinoma arising from the cystic papillary neoplasm was found in the adjacent bile duct and periductal tissue, and these carcinoma cells had also infiltrated into the hepatic parenchyma (Figure 2(e)). Interestingly, we noted peribiliary glands and their conduits near the tumor. Mucin detected by PAS after diastase digestion was observed in the cytoplasm of the dysplastic and invasive carcinoma cells, in addition to mucin secretion within the cyst.

### 2.4. Immunohistochemistry

Immunohistochemical staining for MUC1 (clone Ma695; Novocastra Laboratories, Newcastle, UK), MUC2 (clone Ccp58; Novocastra), MUC5AC (clone CLH2; Novocastra), MUC6 (clone CLH5; Novocastra), CK7 (clone OV-TL 12/30; Dako Inc., Carpinteria, CA, USA), CK20 (clone Ks 20.8; Dako), S100P (EPR6143a; Abcam, San Francisco, USA), and CDX2 (DAK-CDX-2) was performed using the EnVision+ system (Dako). In Case  1, the dysplastic and carcinoma cells were strongly positive for CK7, MUC1, and MUC6 (Figures 1(e) and 1(f)) but negative or only focally positive for CK20, MUC2, CDX2, and MUC5AC. These findings were compatible with the PB type with oncocytic foci in consideration of the histological features. In Case  2, the neoplastic and carcinoma cells were strongly positive for MUC5AC and MUC6 (Figure 2(f)) but only focally positive or negative for MUC1, MUC2, CDX2, CK7, and CK20, suggesting that this immunophenotype was compatible with the gastric type.

In both cases, the peribiliary glands and their conduits were positive for MUC6 (Figure 1(g)) in the background liver, but the bile duct lining cells were only focally positive or negative for MUC6.

Neither estrogen nor progesterone receptor-positive stromal cells were found in the cyst walls in either case.

## 3. Discussion

The clinicopathological findings of these two cases are summarized as follows: (i) solitary cystic neoplasm at the hepatic hilum showing no communication with the adjacent bile ducts, (ii) well-differentiated papillary neoplasm composed of high-grade dysplasia with fine fibrovascular stroma growing in the cysts and associated with an invasive carcinoma, (iii) a capsule-like wall surrounding the tumor without ovarian-like stroma, and (iv) peribiliary glands around the tumor and high-grade neoplastic and carcinoma cells positive for MUC6, which was constitutively expressed in the nonneoplastic peribiliary glands but not in the epithelia lining the bile duct.

Cystic neoplasms of the liver are classifiable into MCN or cystic IPNB according to the 2010 WHO classification [2, 3]. However, the cases reported here differed from these neoplasms, as there was no communication between the cystic tumors and the adjacent bile duct lumen nor any ovarian-like stroma expressing estrogen or progesterone receptors in the capsule or stroma of these tumors.

The peribiliary glands are located in the connective tissue around the perihilar and extrahepatic large bile duct and drain into the bile duct lumen via their own conduits [1, 15]. Neoplasms, including carcinoma and dysplastic papillary neoplasm, reportedly occur in the bile duct lumen as well as the peribiliary cysts [2, 7, 16]. The present cases were located near the hepatic hilum adjacent to the hilar bile ducts, where the peribiliary glands are densely distributed [17]. The peribiliary glands were close to these cystic neoplasms, raising the possibility that these tumors might have originated from these glands or their conduits. Sasaki et al. reported that the peribiliary glands and their conduits strongly expressed MUC6, while the lining epithelium of the bile ducts did not [18]. Our cases expressed MUC6 strongly in high-grade dysplastic cells and invasive carcinoma cells. Interestingly, the peribiliary glands and conduits were positive for MUC6, while the epithelia lining the adjacent bile ducts were negative, similar to the results of Sasaki et al. [18]. The case reported by Nakanishi et al. was a cystic papillary neoplasm probably arising from the peribiliary glands that showed communication with the adjacent bile duct and strong expression of MUC6 [12]. Given these present and previous findings, we considered that the tumors in the present case might have originated from the peribiliary glands, but communication with the bile duct was lost during the development and progression of these neoplasms.

IPNB is eventually followed by invasive cholangiocarcinoma [2–4]. Almost all IPNB cases reported thus far have involved the lining epithelia of the bile ducts, with the affected bile ducts showing evident and occasionally prominent dilatation, and the tumor was located in the dilated lumen [2–4]. IPNB was originally proposed as a counterpart of intraductal papillary mucinous neoplasm (IPMN) of the pancreas [2, 19–21]. IPMN is classified into the main duct type and branch type, and the IPNB cases reported thus far appear to correspond to the main duct type of IPMN [22]. Nakanishi et al. suggested that papillary cystic neoplasms arising from the peribiliary glands may correspond to the branch type of IPNB [8]. Interestingly, the cases reported here appeared to originate from the peribiliary gland and thus may correspond to the branch type of IPNB, a counterpart of the branch type of IPMN.

**Figure 3:**
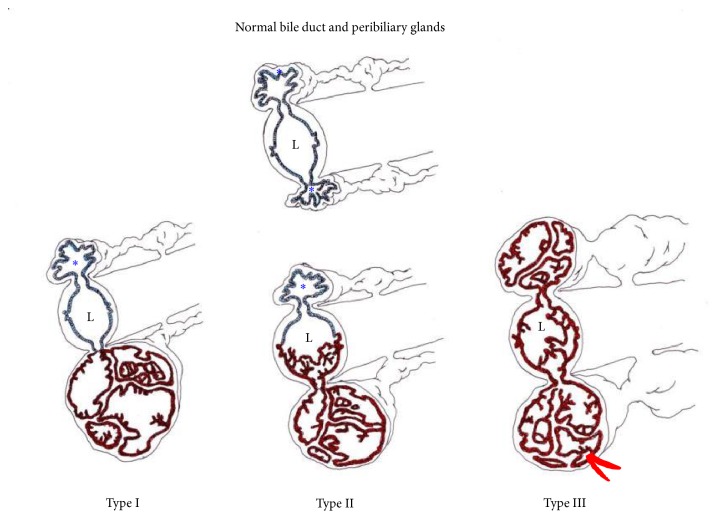
A schematic diagram of cystic and papillary neoplasms involving the peribiliary glands (PGs). The blue epithelium indicates nonneoplastic glands, and brown epithelium indicates neoplastic glands. Red indicates an invasion of carcinoma arising in the neoplasm of the PGs. L: bile duct lumen; ^*∗*^ unaffected PGs. Type I shows neoplastic involvement confined to the PG, and type II shows main involvement of the PGs with partial secondary involvement of the adjacent bile duct. Type III reflects extensive involvement of the PGs with simultaneous involvement of the bile ducts, which may appear grossly as peribiliary cysts in the perihilar regions.

There have been several reports suggesting that IPNB cases involve the peribiliary glands and then spread to the bile duct lumen [7, 8, 11, 12]. The cystic tumors of Case  1 in the present study and of the case reported by Nakanishi et al. [8] were mainly localized around the hilar bile ducts, and neoplastic cells were located within the cystic lesions. Case  1 showed focal capsular invasion of carcinoma, and Case  2 showed evident involvement of the adjacent right hepatic and posterior sectional ducts by invasive carcinoma arising from IPNB. The development and progression of IPNB originating from the peribiliary glands and its relationship to the adjacent bile ducts are schematically shown in Figure 3 [7]: Case  1 may correspond to type 1 and Case  2 to type 2. Regarding the differentiation of branch type IPNB showing intraluminal spread to the adjacent bile ducts, particularly type III, from secondary involvement of the peribiliary glands by CCA arising from the epithelia lining the bile duct, the latter usually does not show gross cystic changes of the peribiliary glands, according to our routine experience. However, accumulation of more cases will be required to validate the precise spread of IPNB arising from the peribiliary glands.

In conclusion, we reported two cases of biliary neoplasm morphologically characterized by an intracystic papillary neoplasm composed of diffuse high-grade dysplasia with fine fibrovascular cores and with an associated invasive carcinoma at the hepatic hilum. The lesions in these cases, which were not associated with ovarian-like stroma and had no communication with the bile duct lumen, might have been derived from the peribiliary glands around the bile ducts. These two cases constitute a subtype of cystic papillary biliary neoplasms derived from the peribiliary glands and can be referred to as branch type IPNB.

## Supplementary Material

The cystic papillary tumor shows intraepithelial infiltration through the peribiliary glands and conduits around the tumor, but does not reach the bile duct lumen.
