# Supplementary figures and images for: Cystic and Papillary Neoplasm at the Hepatic Hilum Possibly Originating in the Peribiliary Glands
Source: Case Rep Pathol. 2016 Aug 31;2016:9130754. doi: 10.1155/2016/9130754 (PMC5021872; doi:10.1155/2016/9130754)

## Slide 1
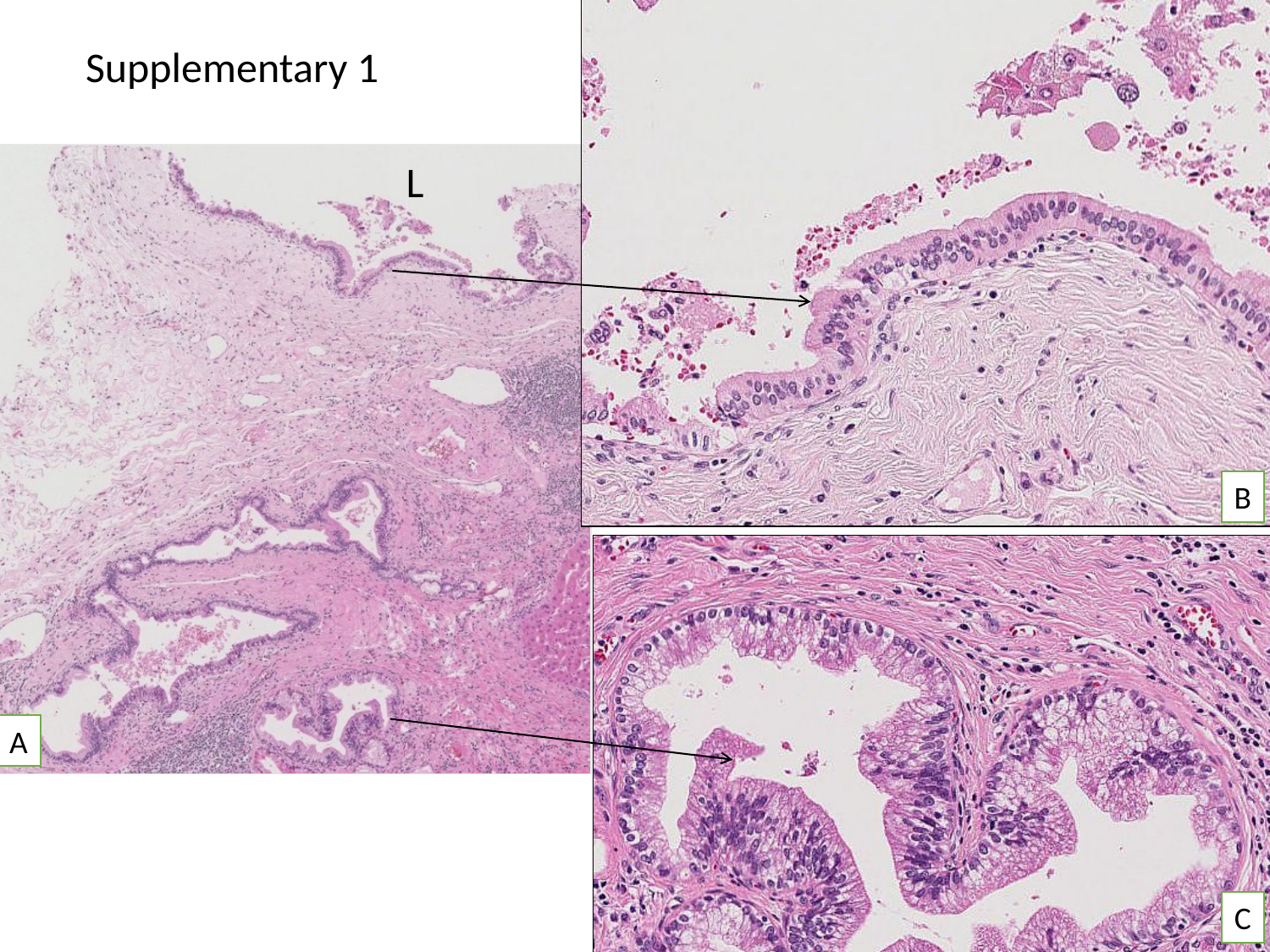

Supplementary 1
L
B
↓
A
C

Supplement: Supplementary file 1 — The cystic papillary tumor shows intraepithelial infiltration through the peribiliary glands and conduits around the tumor, but does not reach the bile duct lumen. [file 9130754.f1.pptx]
